# Dietary Intervention Restored Menses in Female Athletes with Exercise-Associated Menstrual Dysfunction with Limited Impact on Bone and Muscle Health

**DOI:** 10.3390/nu6083018

**Published:** 2014-07-31

**Authors:** Lynn Cialdella-Kam, Charlotte P. Guebels, Gianni F. Maddalozzo, Melinda M. Manore

**Affiliations:** 1 School of Medicine, Department of Nutrition, Case Western Reserve University, WG 48, 2109 Aldebert Rd., Cleveland, OH 44106-4954, USA; E-Mail: lynn.kam@case.edu; 2 Tahoe Forest Hospital, 10121 Pine Ave, Truckee, CA 96161, USA; E-Mail: cguebels@tfhd.com; 3 School of Biological and Population Sciences, Nutrition and Exercise Science, Oregon State University, 103 Milam Hall, Corvallis, OR 97331, USA; E-Mail: gianni.maddalozzo@oregonstate.edu

**Keywords:** amenorrhea, oligomenorrhea, energy intake, energy expenditure, exercise performance, bone health, protein synthesis, protein degradation, muscle strength

## Abstract

Exercise-related menstrual dysfunction (ExMD) is associated with low energy availability (EA), decreased bone mineral density (BMD), and increased risk of musculoskeletal injury. We investigated whether a 6-month carbohydrate-protein (CHO-PRO) supplement (360 kcal/day, 54 g CHO/day, 20 g PRO/day) intervention would improve energy status and musculoskeletal health and restore menses in female athletes (*n* = 8) with ExMD. At pre/post-intervention, reproductive and thyroid hormones, bone health (BMD, bone mineral content, bone markers), muscle strength/power and protein metabolism markers, profile of mood state (POMS), and energy intake (EI)/energy expenditure (7 day food/activity records) were measured. Eumenorrheic athlete controls with normal menses (Eumen); *n* = 10) were measured at baseline. Multiple linear regressions were used to evaluate differences between groups and pre/post-intervention blocking on participants. Improvements in EI (+382 kcal/day; *p* = 0.12), EA (+417 kcal/day; *p* = 0.17) and energy balance (EB; +466 kcal/day; *p* = 0.14) were observed with the intervention but were not statistically significant. ExMD resumed menses (2.6 ± 2.2-months to first menses; 3.5 ± 1.9 cycles); one remaining anovulatory with menses. Female athletes with ExMD for >8 months took longer to resume menses/ovulation and had lower BMD (low spine (ExMD = 3; Eumen = 1); low hip (ExMD = 2)) than those with ExMD for <8 months; for 2 ExMD the intervention improved spinal BMD. POMS fatigue scores were 15% lower in ExMD *vs.* Eumen (*p* = 0.17); POMS depression scores improved by 8% in ExMD (*p* = 0.12). EI, EA, and EB were similar between groups, but the intervention (+360 kcal/day) improved energy status enough to reverse ExMD despite no statistically significant changes in EI. Similar baseline EA and EB between groups suggests that some ExMD athletes are more sensitive to EA and EB fluctuations.

## 1. Introduction

Optimal energy intake (EI) and nutrition can improve exercise performance and maintain overall health in physically active individuals [1]. Female athletes, however, can find it difficult to meet energy and nutrient needs while maintaining a low fat or body weight considered optimal for sports performance. Thus, they often restrict EI to make weight goals [1]. Low EI, combined with high levels of exercise, increases the risk of developing exercise-related menstrual dysfunction (ExMD) and poor bone health [2,3].

ExMD can be high in physically active women, ranging from 0% to 60% [4], and occurs across a continuum from mild disruptions in menses (no ovulation or luteal phase deficiency) to oligomenorrhea (cycles ≥ 35 day) and amenorrhea (no menses for >90 day [2]. Low energy availability (EA) (*i.e.*, energy remaining for body functions after exercise training) may lead to menstrual dysfunction through a leptin-controlled pathway [2]. In ExMD, females have low leptin levels that contribute to the disruption of luteinizing hormone (LH) pulsatility via interference of gonadotropin-releasing hormone (GnRH) pulsatile [5]. Sequentially, the ovaries decrease production of estrogen and progesterone, the hormones responsible for triggering the lining and egg of the uterus to be shed (menstruation) resulting in abnormal menses [5].

ExMD is also associated with compromised bone health, including low bone mineral density (BMD, prevalence = 20%–50%), osteoporosis (prevalence = 10%–13%), and stress fractures [2,3,6]. For premenopausal women, low BMD is defined as 2.0–2.5 standard deviation (SD) below the mean BMD of young adults, and osteoporosis is defined as a BMD > 2.5 SD below the mean. For athletes, BMD is typically 5%–15% higher than non-athletes, thus, a BMD > 1.0 SD below the mean may indicate poor bone health [2].

In addition, ExMD has been linked to other health consequences, such as cardiovascular dysfunction, endothelial dysfunction, abnormal metabolic hormonal profile (decreased thyroid, leptin and IFG-1; increased cortisol) and muscle injuries [3,7]. The impact of ExMD on skeletal muscle strength and power, however, has not yet been examined. Chronically low estrogen observed in ExMD could potentially cause muscle damage and alter the muscle repair process, thus prohibiting an athlete from reaching their highest level of sports performance. Estrogen in the skeletal muscle of rodents decreases muscle damage and accelerates repair by possibly acting as antioxidant, cell membrane stabilizer, and/or satellite cells stimulator [8,9]. In young physically active women, the research findings are mixed. Lower estrogen status in women has been reported to be associated with greater muscle damage and oxidative stress after running [10,11] and slower muscle strength recovery after a stretch-shortening exercise load (*i.e.*, 100 drops jumps from platform) [12] but not with exercise-induced muscle damage following either stretch-shortening exercise load [12] or eccentric exercise [13]. Thus, low estrogen status may negatively impact muscle health and strength, but more research in humans is needed [8].

In ExMD, low estrogen levels occur in conjunction with low EA, which potentially could lead to impairment of post-exercise protein metabolism. Miller *et al.* [14] compared myofibrillar protein synthesis in the follicular phase (*i.e.*, low estrogen phase) and luteal phase (*i.e.*, high estrogen phase) of the menstrual cycle in eumenorrheic physically active women and reported no difference in protein synthesis between phases. The women in this study, however, had normal cyclic variation in estrogens and not chronically low estrogen status as in ExMD. To our knowledge, protein metabolism in ExMD has not been examined and therefore, an aim of this study was to assess post-exercise indicators of protein metabolism (*i.e.*, AMPK, p70S6K, and FOXO1).

Oral contraceptives (OCs) are often prescribed to restore menses and prevent bone loss in ExMD, but their impact on bone health is equivocal [15]. In addition, OCs do not restore non-reproductive hormone levels (e.g., leptin, IFG-1, thyroid, cortisol) and can have undesirable side effects such as weight gain, fatigue, mood disorders, nausea, and headaches [16]*.* Currently there are no long-term interventions examining non-pharmacological treatments of active females with ExMD [17], thus, an alternative lifestyle approach, such as one that alters diet or physical activity is desirable. Based on our previous research [18], we hypothesized that an increase in EI (+360 kcal/day) would improve EB and bone health and restore reproductive function in ExMD. In addition, we hypothesized that muscle strength and power in the ExMD group would increase with the restoration of normal estrogen levels and EB improvements from the intervention. Therefore, the purpose of this 6-month intervention was to determine if increases in EI, using a daily carbohydrate-protein (CHO-PRO) supplement (360 kcal/day), would improve EB, bone health, muscle strength and power, and mood state, and restore reproductive function in physically active women with ExMD. A secondary aim was to compare diet, indicators of protein metabolism, hormones, and bone biomarkers between women with ExMD and eumenorrheic (Eumen) physically active controls.

## 2. Experimental Section

### 2.1. Study Protocol and Participants

**Figure 1:**
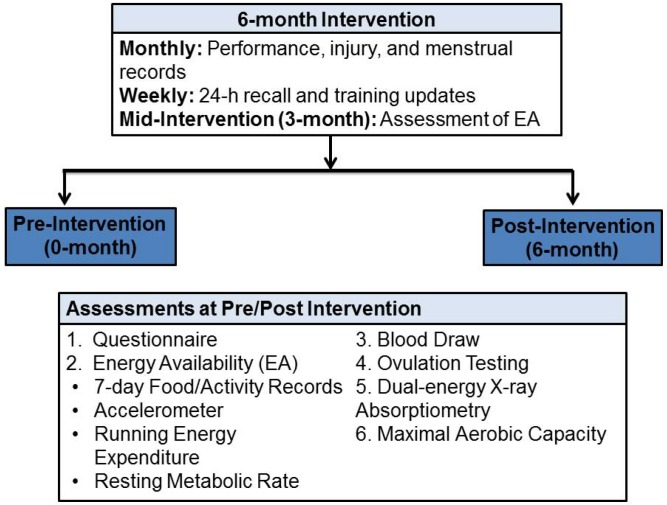
Detailed Protocol of Study: Women with exercise-induced menstrual dysfunction (ExMD, *n* = 8) were assessed at pre (0-month)/post (6-months)-intervention; a mid-intervention (3-months) assessment was done to monitor compliance. ExMD were compared at pre/post-intervention to a group of Eumenorrheic athletes (*n* = 10), who were assessed at 0-month only.

Endurance trained women (12 amenorrheic/oligomenorrheic [ExMD]; 10 Eumen) were recruited. Overall, 8 of 12 women with ExMD completed the intervention; 4 women dropped out due to personal reasons. An ExMD control group was not included due to ethical reasons (*i.e.*, no treatment for 6–12-months); the University Institution Review Board (IRB), which reviews and approves research involving human subjects, would only allow tracking ExMD participants that choose not to complete the intervention after the initial screening (none completed this option). After obtaining IRB approved informed consent, all participants completed baseline questionnaires to assess general health, exercise training, and menstrual/dietary history (0-month; Figure 1). Two subscales (Drive for Thinness and Body Dissatisfaction) of the Eating Disorder Inventory-2 (EDI-2) questionnaire [19] were also administered to identify athletes with disordered eating [20]. At pre/post-intervention, mood state was assessed using a Profile of Mood State (POMS) questionnaire [21], which measures 6 different mood states (fatigue, anger, vigor, depression, confusion, anxiety). A total mood disturbance score (maximum = 60) was calculated to provide an overall measure of global effective state. Inclusion criteria included ≥7 h/week of exercise training for the last 2 years, a VO_2_max > 38 mL/kg/min, no OCs or hormonal replacement therapy use for 6-months, EDI-2 subscale score <14, and no self-reported primary amenorrhea or non-exercise-related amenorrhea.

Participants were assigned to either the ExMD (*n* = 8) or Eumen (*n* = 10) group based on self-reported menstrual history and ovulation status (Clearblue^®^ Easy Fertility Monitor, Waltham, MA, USA), which the participants measured daily for ≥1 month. Participants who began menses during the intervention tested for ovulation each month until the study ended. Other types of menstrual dysfunction were eliminated based on assessments of LH, follicular stimulating hormone (FSH), prolactin levels, and LH/FSH ratio [2,22]. All participants had normal thyroid (T_3_) levels. ExMD group was assessed at 0-month/6-months; Eumen group at 0-month only (Figure 1).

### 2.2. Maximal Aerobic Capacity Test (VO_2_max)

Participants completed a standardized treadmill VO_2_max test using indirect calorimetry (ParvoMedics Metabolic Cart, Sandy, UT, USA) as previously reported [23].

### 2.3. Blood Biochemistry

Fasting blood was drawn for a general blood screen, including iron and vitamin (B-12, folate, 25-OH Vitamin D) status, T_3_ and reproductive hormones (estradiol, LH, FSH, prolactin, progesterone) (Samaritan Health, Corvallis, OR, USA). The rationale for assessing the micronutrients and hormones selected above are as follows: (1) Female athletes are at risk for deficiency of vitamins B12 and folate due to inadequate dietary intake and/or increased needs [24]; (2) Active women, especially endurance athletes, are at risk for low iron [1] and vitamin D status [25]; (3) With menstrual dysfunction, T_3_ and reproductive hormones are typically evaluated [3]. In addition, serum osteocalcin, a bone-specific protein of osteoblasts, and Procollagen Type I Intact *N*-Terminal Propeptide (P1NP), an indicator of newly formed type I collagen, were assessed as indicators of bone formation [26]. Carboxyterminal telopeptide of type I collagen (CTX) was used as a marker of bone resorption [26]. These bone markers (osteocalcin, P1NP, and CTX) were measured using ELISA (CV: Osteocalcin = 2.5%; P1NP = 2.9%; CTX = 3.3%) (Immunodiagnostics Systems, Scottsdale, AZ, USA).

### 2.4. Energy Intake and Expenditure Measurement and Analysis

#### 2.4.1. Measurements

For 7-consecutive days participants complete weighed food records, physical activity (PA) logs and wore an accelerometer (ActiGraph LLC, Pensacola, FL, USA). To improve accuracy of energy expenditure (EE) and total energy expenditure (TEE) estimates, running energy expenditure (RunEE) and resting metabolic rate (RMR) were measured using indirect calorimetry [23]. Briefly, RunEE was measured as participants ran for 5-min at 4 self-selected speeds ranging from easy (warm-up) to fast (5 K race pace). RMR was measured on 2 separate days within a 7 day period (8-h fast; ~19 h since last exercise).

#### 2.4.2. Analysis

As previously reported [23], TEE was calculated as the sum of three components as outlined by Tomten and Hostmark [27]: RMR, all PA expenditures (activities of daily living plus EEE from 7 day activity logs), and the thermic effect of food. RunEE data was used to estimate each participant’s EEE during running. Diet and PA records were analyzed using a nutrient and activity analysis program (Food Processor SQL, ESHA Research, Salem, OR, USA). Under-reporters were identified using Goldberg *et al.* [28] criteria and one Eumen control was excluded from the dietary analysis. EB and EA were then calculated as follows: EB (kcal/day) = EI-TEE; EA (kcal/day) =EI-EEE. Exercise was defined as PA > 4.0 metabolic equivalents (METs) to eliminate activity of daily living (*i.e.*, bicycle commute) from EEE calculation.

### 2.5. Bone Density and Body Composition

Dual-energy X-ray absorptiometry (DXA, Hologic QDR-4500 Elite A; Waltham, MA, USA) was used to measure whole body composition (bone, muscle, and fat mass) and areal BMD of the proximal femur (total hip, femoral neck, greater trochanter) and the lumbar spine (L1–L4). All scans were performed and analyzed by a trained technician with over 20 years of experience in bone density and body composition assessment (CV = 1.5% and 1.0% for whole body, and hip and lumbar spine BMD). Based on *z*-scores, BMD were classified as having normal BMD for *z*-score > −1.0, low BMD for *z*-scores between −1.0 and −2.5, and osteoporosis for *z*-scores < −2.5 [29].

### 2.6. Muscle Strength and Power

Isokinetic concentric strength (peak power (watts) and torque (Nm)) of knee extension and flexion, and plantar and dorsiflexion were measured at speeds of 60, 90 and 120 degrees/s with gravity corrected using a validated and reliable mechanical system, the Biodex System 3 [30] (Biodex Medical Systems, Inc. Shirley, NY, USA). After being familiarized with the test, participants perform 10 trials of each exercise at low intensity to warm-up followed by three sets of 10 maximal efforts (one set at each speed) to determine peak torque (Nm) and power (watts). Each maximal effort was separated by ~60 s of rest. Strength assessment protocols were programmed into the dynamometer to set parameters for testing (*i.e.*, start and stop angles and speed of contraction), and therefore ensured consistency. These protocols have good reliability within this population (CV = 4% to 8%). The dynamometer software (Biodex System 3 Advantage Software; Biodex Medical Systems Inc., Shirley, NY) generated peak power (watts) and torque (Nm) for each speed measured. The Bassey Power Rig (Medical Engineering Unit, University of Nottingham School of Biomedical Sciences, Nottingham, England) was used to assess explosive muscular power of the lower extremities as previously described [31]. Explosive power is a term used to describe the muscle’s ability to perform work in ≤0.5 s [31]. Participants performed a 5-min warm-up consisting of walking on the treadmill. The testing procedure required 10 maximal leg presses on each leg with their knee starting at a 90 degree angle. The participants were asked to press down on the push pedal one leg at a time to full extension, alternating right and left leg. The highest and lowest measurements were not used. The average of the remaining 8 measurements was used to calculate peak torque and force. Muscle strength and power assessments were conducted by trained researcher under supervision of exercise physiologist with over 20 years of experience in these measurements.

### 2.7. Submaximal Exercise Protocol and Skeletal Muscle Analysis

#### 2.7.1. Submaximal Exercise and Post-Exercise Muscle Biopsy

After an 8-h fast, the participants arrived to the laboratory and completed a 45-min run at 75% of VO_2_max. After completing the run, the participants were provided a CHO-PRO nutrition shake (Gatorade^®^ Nutrition Drink; 325 mL; 360 kcal) to consume immediately after exercise. At 60-min post-exercise, a muscle biopsy was obtained from vastus lateralis by the percutaneous needle biopsy technique. First, the thigh was disinfected using an antiseptic/antimicrobial cleanser (chlorhexidine gluconate) and anesthetized with an injection of mercaine and lidocaine. A small incision, ~3/8 in long, was made through the skin and the fascia. Pressure was applied immediately to stop any bleeding prior to taking the biopsy. Suction was applied to maximize sample size. The biopsy was trimmed of adipose tissue and frozen in liquid nitrogen at −80°C for subsequent analysis.

#### 2.7.2. Muscle Biopsy Preparation and Antibodies and Positive Controls

Muscle samples (~40 mg) were homogenized in RIPA buffer (Cell Signaling, Beverly MA) and a DC protein assay (Bio-Rad Laboratories, Hercules, CA, USA) was used to determine protein concentration. Monoclonal antibodies were used for phospho-p70S6k (Thr389), total p70S6k, phospho-AMPKα (Thr172), and total AMPKα; Polyclonal antibodies were used for phospho-FOXO1 (Ser256) and total FOXO1 (Cell Signaling, Beverly, MA, USA). An anti-rabbit IgG, HRP-linked antibody was used for all western blots (Cell Signaling, Beverly, MA, USA). Positive controls were loaded for AMPK and p70S6k analysis.

#### 2.7.3. Western Blotting 

The muscle homogenates (70 μg·μL^−1^) were diluted in a sample buffer mixture (6.25 μL of 4× Sample Loading buffer and 2.5 μL of 10× Reducing Agent (Invitrogen, Carlsbad, CA, USA)), and deionized water and then boiled for 5 min at 90 °C. Equal amounts of proteins (55 μg/lane) were loaded into NuPAGE^®^ 4%–12% Bis Tris Gel and ran for 90-min at 120 volts at room temperature (RT). Proteins were then transferred to nitrocellulose membrane (35 volts, 60-min). Membranes were blocked in 5% nonfat dry milk (NFDM) Tris-Buffered Saline with 0.1% Tween-20 (TBST) for 30-min on a rocker at RT. The membranes were incubated in 1:1000 dilution of primary antibody in 5% BSA TBST overnight at 4 °C on a rocker. The membranes were then washed 3 times for 5-min and incubated in 1:3000 dilution of secondary antibody in 5% NFDM TBST for 60-min on a rocker at RT. The membranes were washed again in TBST (3×/5-min) and incubated in chemiluminescent substrates (Thermo Fisher Scientific, Rockford, IL, USA) for 2-min on a rocker at RT. A digital image of the membranes was taken on FluorChem TM 8900 (Cell Biosciences, Santa Clara, CA, USA), and quantification was performed using ImageJ (NIH, Bethesda, MD, USA). After visualization, the membranes were stained with Ponceau S to verify equal loading. The outcome measures were phosphorylated AMPK (% of total AMPK normalized to control) as a marker of energy homeostasis, phosphorylated p70S6k (% of total p70S6K) as a marker of protein synthesis, and phosphorylated FOXO1 (% of total FOXO1) as a marker of protein degradation. Analysis of Western blots was completed by a trained researcher under the supervision of a muscle physiologist with over 20 years of experience in evaluating this type of assay.

### 2.8. Intervention Assessments

After baseline assessments, the ExMD participated in a 6-month CHO-PRO supplement intervention, in which they added 325 mL of Gatorade^®^ Nutrition Drink (360 kcal·day^−1^, 54 g carbohydrate, 20 g protein, 8 g fat, 300 mg of calcium, 100 IU of vitamin D, 0.4 mg of vitamin B6, and 1.2 µg of vitamin B12) to their daily diets. The participants consumed the supplement 30–60 min post-exercise or when convenient on non-exercise days. Participants exercised ~6–7 day/week. Researchers met with participants weekly to ensure compliance, provide supplement supply, discuss issues, and complete a 24-h dietary recall. Participants were asked to return empty cans to weekly meetings and also asked if they consumed the supplement as instructed. Additionally, to assess compliance, energy status (*i.e.*, EI and TEE as described previously) was measured at 3-months. Performance, injury, and menstrual cycle diary were collected monthly.

### 2.9. Statistical Analysis

Power calculations indicated a sample size of 8/group (~90% power; α = 0.01) was required to detect a significant improvement in EB of 360 kcal/day, based on earlier research [18]. The data were summarized using means ± SD. For demographics variables and dietary measures, simple linear regression was used to compare groups and ExMD pre/post-intervention blocking on participant. For BMD, BMC, blood bone biomarkers/hormones, and skeletal muscle measures, comparisons between groups and pre/post-intervention blocking on participant were made using multiple linear regression (MLR) and extra-sums-of-squares F-tests. Age and weight at baseline were controlled for in MLR regressions. Outliers were assessed using Cook’s Distance and Studentized Residuals. Adjusted *p*-values were calculated to control for multiple comparisons at a false discovery rate (FDR) of 5%. All statistical analysis was done using S-plus (TIBCO, Palo Alto, CA, USA). Statistical significance was set at *p* < 0.05.

## 3. Results 

### 3.1. Physical Characteristic

Eumen and ExMD were similar in age, menarcheal age, body composition, VO_2_max, BMD, and BMC at 0/6-months (Table 1 and Table 2). With the intervention, trends for increased mean body weight, body fat, and BMI were observed but were not significant (*p* ≥ 0.11). No changes in LBM, FFM, VO_2_max, BMC, and BMD were observed.

### 3.2. Energy Availability, Energy Balance, and Energy and Nutrient Intakes

At 0/6-months, EI, EB, and EA were similar between groups (Table 2). Important physiological improvements in EI, EB and EA, associated with mean weight gain of ~1.6 kg, were observed with the intervention. Weight gain was observed in 75% (6/8) of the ExMD women. The mean increase in EI in this group was similar to the energy content of the supplement (360 kcal/day). Overall, 62% (5/8) and 75% (6/8) of the ExMD women had a positive increase in EB and EA, respectively. In the ExMD group, EA was <30 kcal/kg FFM/day for 3 women at baseline and 1 at post-intervention.

Overall nutrient intakes for both groups were adequate and did not change with the intervention (Table 3). Mean protein intake was within the recommended intake for physically active individuals (1.2–1.7 g/kg) [1], and mean fiber intake was above the adequate intake (AI) recommendation of 25 g/day. Overall, 38% (3/8) of ExMD and 40% (4/9) of Eumen used vitamin/mineral supplements at baseline, while only one ExMD use supplements at post-intervention. Most of the Eumen and all ExMD had adequate dietary intake (≥Estimated Average Requirement (EAR)) for vitamin B12, calcium, iron, phosphorous, and zinc. For the ExMD group, inadequate baseline dietary intake (<EAR) was observed for folate at 38% (3/8), vitamin B12 at 13% (1/8), and vitamin D at 63% (5/8) with no change post-intervention. For the Eumen group, low dietary intakes were found for folate (11% (1/9)), vitamin B12 (11% (1/9)), and vitamin D (56% (5/9)).

**Table 1:** Physical characteristics in athletes classified as eumenorrheic (Eumen) or with exercise-induced menstrual dysfunction (ExMD) before and after a 6-month dietary intervention ^a–c^.

Values expressed as Mean ± SD	Eumenorrheic Controls (*n* = 9)	Women with ExMD (*n* = 8)	
Description	0-months	0-months	6-months
Age (year)	23.1 ± 4.3	22.6 ± 3.3	-
Age at Menarche (year)	12.7 ± 1.3	13.5 ± 2.0	-
Weight (kg)	66.8 ± 9.3	62.4 ± 7.8	64.0 ± 8.0
Lean Body Mass (kg) ^d^	48.5 ± 4.7	46.2 ± 4.4	46.1 ± 4.7
Fat Free Mass (kg) ^d^	51.0 ± 5.0	48.5 ± 4.6	48.4 ± 4.8
Body Mass Index (BMI) (kg/m^2^)	23.2 ± 2.8	22.3 ± 2.5	22.9 ± 2.5
Body Fat (%) ^d^	23.2 ± 4.4	22.0 ± 4.7	24.1 ± 3.9
Exercise (h/week) ^e^	7.4 ± 3.6	7.4 ± 3.2	7.1 ± 3.4
VO2max (mL/kg/min) ^f^	50.6 ± 5.2	49.0 ± 5.8	49.3 ± 6.0
VO2max (L/min) ^f^	3.3 ± 0.4	3.0 ± 0.3	3.1 ± 0.4

^a^ The women with ExMD participated in a 6-month carbohydrate-protein supplement intervention (360 kcal/day)). Eumen were measured at baseline (0-month) only and compared to ExMD at 0-months and at 6-months; ^b^ No significant differences were detected (*p*-value > 0.05 for all comparisons). Adjusted *p*-values were calculated to control for multiple comparisons at a FDR of 5%; ^c^ Dietary intake includes any vitamin and mineral supplements and includes the daily consumption of the CHO-PRO supplement at 6-months for ExMD; ^d^ Measurements were made using DXA; ^e^ Exercise was defined as physical Activity >4.0 METS; ^f^ VO_2_max = maximal aerobic capacity.

**Table 2:** Energy availability (EA), energy balance (EB), bone mineral content (BMC), and bone mineral density (BMD) in athletes classified as eumenorrheic (Eumen) or with exercise-induced menstrual dysfunction (ExMD) before and after a 6-month dietary intervention ^a–c^.

Values expressed as Mean ± SD	Eumenorrheic Controls (*n* = 9)	Women with ExMD (*n* = 8)	
Description	0-months	0-months	6-months
*Bone Mineral Content (g) ^d^*			
Whole Body	2492 ± 332	2326 ± 314	2331 ± 280
Total Hip	39 ± 6	33 ± 8	34 ± 8
Total Spine	66 ± 13	61 ± 14	61 ± 13
*Bone Mineral Density (g·cm^2^) ^d^*			
Whole Body	1.2 ± 0.1	1.2 ± 0.1	1.2 ± 0.1
Total Hip	1.1 ± 0.1	1.0 ± 0.2	1.0 ± 0.2
Total Spine	1.1 ± 0.1	1.0 ± 0.2	1.0 ± 0.1
Energy Intake (kcal/day)	2430 ± 524	2312 ± 324	2694 ± 541
Resting Metabolic Rate (kcal/day)	1491 ± 117	1514 ± 142	1522 ± 134
Energy Balance (kcal/day)	−171 ± 459	−510 ± 361	−44 ± 707
Energy Balance (kcal/kg FFM/day)	−3.0 ± 9.7	−10.3 ± 6.9	−0.7 ± 15.1
Energy Availability (kcal/day)	1945 ± 452	1760 ± 429	2177 ± 645
Energy Availability (kcal/kg FFM/day) ^e^	38.3 ± 10.3	36.7 ± 10.2	45.4 ± 14.7

^a^ The women with ExMD participated in a 6-month carbohydrate-protein (CHO-PRO) supplement intervention (360 kcal/day). Eumen were measured at baseline (0-month) only and compared to ExMD at 0-month and at 6-months; ^b^ No significant differences were detected (*p*-value > 0.05 for all comparisons). Adjusted *p*-values were calculated to control for multiple comparisons at a FDR of 5%; ^c^ Dietary intake includes any vitamin and mineral supplements and includes the daily consumption of the CHO-PRO supplement at 6-months for ExMD; ^d^ Measurements were made using DXA; ^e^ Exercise was defined as physical Activity > 4.0 METS.

**Table 3:** Nutrient intakes in athletes with eumenorrheic (Eumen) or exercise-induced menstrual dysfunction (ExMD) before and after a 6-month dietary intervention ^a–c^.

Values Expressed as Mean ± SD	Eumenorrheic Controls (*n* = 9)	Women with ExMD (*n* = 8)	
Description	0-months	0-months	6-months
*Carbohydrate*			
% of Total Energy	50 ± 5	53 ± 7	51 ± 6
g/day	304 ± 69	308 ± 56	340 ± 42
g/kg/day	4.6 ± 1.0	5.0 ± 1.2	5.4 ± 0.4
*Protein*			
% of Total Energy	15 ± 3	15 ± 4	17 ± 2
g/day	89 ± 21	87 ± 17	114 ± 27
g/kg/day	1.3 ± 0.3	1.4 ± 0.2	1.8 ± 0.5
*Fat*			
% of Total Energy	34 ± 5	30 ± 3	30 ± 5
g/day	93 ± 27	76 ± 16	93 ± 33
*Alcohol*			
% of Total Energy	2 ± 3	3 ± 4	3 ± 4
g/day	9 ± 10	11 ± 14	11 ± 14
Fiber (g/day)	29 ± 11	28 ± 9	26 ± 9
*Vitamins and Minerals*			
Folate (μg/day)	449 ± 207	532 ± 468	403 ± 242
Vitamin B12 (μg/day)	8 ± 5	14 ± 25	6 ± 2
Vitamin D (IU/day)	385 ± 314	379 ± 321	383 ± 316
Calcium (mg/day)	1211 ± 385	1320 ± 571	1725 ± 555
Iron (mg/day)	24 ± 9	29 ± 15	22 ± 5
Magnesium (mg/day)	365 ± 194	288 ± 115	330 ± 69
Phosphorous (mg/day)	1098 ± 471	911 ± 384	1034 ± 386
Zinc (mg/day)	14 ± 6	13 ± 8	16 ± 5

^a^ The women with ExMD participated in a 6-month carbohydrate-protein (CHO-PRO) supplement intervention (360 kcal/d). Eumen were measured at baseline (0-month) only and compared to ExMD at 0-month and at 6-months. One eumenorrheic participant was excluded due to underreporting; ^b^ No significant differences were detected (*p*-value > 0.05 for all comparisons). Adjusted *p*-values were calculated to control for multiple comparisons at a FDR of 5%; ^c^ Dietary intake includes any vitamin and mineral supplements and includes the daily consumption of the CHO-PRO supplement at 6-months for ExMD.

Blood assessments of vitamins (B12, D, and folate) did not differ between groups and were generally normal (Table 4). One ExMD women had elevated blood B12 levels due to high supplementation (100 µg B12/day); one participant had iron deficiency anemia, and 3 participants (Eumen, *n* = 2; ExMD, *n* = 1) had low serum ferritin levels.

**Table 4:** Blood bone markers, hormones levels and ovulation status in athletes classified as eumenorrheic (Eumen) or with exercise-induced menstrual dysfunction (ExMD) before and after a 6-month dietary intervention ^a,b^.

Values expressed as Mean ± SD	Eumenorrheic Controls (*n* = 10)	Women with ExMD (*n* = 8)		NormalValues ^c^
Description	0-months	0-months	6-months	
*Bone Markers ^d^*				
P1NP (ng/mL)	42.0 ± 30.8	52.9 ± 36.4	58.5 ± 19.2	27.7–127.6 ^e^
Osteocalcin (nmol/L)	4.7 ± 1.4	4.5 ± 1.3	4.4 ± 1.7	1.8–7.8 ^e^
CTX (ng/mL)	0.754 ± 0.248	0.661 ± 0.324	0.660 ± 0.214	0.000-0.700 ^e^
*Hormones*				
T (nmol/L)	1.70 ± 0.16	1.61 ± 0.32	1.62 ± 0.24	1.20–2.74
Estradiol (pmol/L)	158.1 ± 115.4	232.6 ± 260.7	399.9 ± 557.8	45.9–609.4
Progesterone (nmol/mL)	1.8 ± 1.1	3.2 ± 3.1	2.5 ± 1.4	0.6–4.8
LH (IU/L)	5.9 ± 3.3	5.6 ± 4.1	15.3 ± 16.3	2.4–12.6
FSH (IU/L)	5.5 ± 1.2	4.1 ± 2.2 ^d^	5.1 ± 2.0	4–13
*Vitamins*				
Folate (ng/mL)	14.9 ± 3.1	16.2 ± 2.8	15.0 ± 2.0	>9.1
B12 (pg/ml)	561 ± 116	704 (381)	689 ± 290	211–946
25-OH Vit D (/mL)	105.4 ± 30.1	106.7 ± 24.6	115.1 ± 19.2	14.7–162.0
*Iron Panel*				
Serum Iron (/dL)	115 ± 58	104 ± 61	74 ± 33	37–145
TIBC (/dL)	313 ± 57	364 ± 62	346 ± 43	250–450
% Saturation	37 ± 18	29 ± 16	22 ± 11	15–50
Ferritin (ng/mL)	36.5 ± 26.0	27.5 ± 16.6	31.2 ± 15.8	13–150
Ovulation ^f^	*n* = 10	*n* = 0	*n* = 7	-

^a^ The women with ExMD participated in a 6-month carbohydrate-protein (CHO-PRO) supplement intervention (360 kcal/day). Eumen were measured at baseline (0-month) only and compared to ExMD at 0-month and at 6-months; ^b^ No significant differences were detected (false discovery rate) *p*-value > 0.05 for all comparisons). Adjusted *p*-values were calculated to control for multiple comparisons at a FDR of 5%; ^c^ Normal ranges reported by hospital at the time of the analysis; ^d^ Procollagen Type I Intact *N*-Terminal Propeptide (P1NP) and osteocalcin are bone formation markers. Carboxy-terminal collagen crosslinks (CTX) is a bone resorption marker. These were measured using ELISA; ^e^ Normal ranges as reported in assay. ^f^ Ovulation represents number of women who test positive for ovulation using Clearblue^®^ Easy Fertility Monitor. Women with ExMD measured ovulation status every day for one month prior to starting the intervention.

### 3.3. Menstrual Status

ExMD participants resumed menses (mean time to 1st period = 2.63 months; range = 1–7 months) and 7 of 8 (88%) were ovulating. For those reporting amenorrhea for <1 year (*n* = 5) *vs.* >1 year (*n* = 2), it took 1–2 months or 6-months, respectively, for menses to resume. Serum thyroid or reproductive hormones did not differ between groups nor change with the intervention (Table 4).

### 3.4. Bone Health

Mean BMD and BMC was similar between groups, but more ExMD women were classified with low hip and spinal BMD. All Eumen women had normal hip BMD, and all but one had normal spinal BMD. At baseline, 2 ExMD women had low BMD at the hip, which did not improve with the intervention. For the spine, two ExMD participants had low BMD; one improved status with the intervention. One ExMD woman had spinal osteoporosis improved status to low BMD by post-intervention. A negative relationship was observed between duration of ExMD and total body BMD (Table 5); women with longer duration of ExMD tended to have lower *z*-scores at the hip and spine. Finally, there were no significant changes in serum bone markers with the intervention; groups were also similar at baseline (Table 4).

**Table 5:** Bone status (BMD *z*-score) in women with ExMD before and after a 6-month dietary intervention by months since last menses ^a^.

Months since Last Menses ^b^	*n* Size	Total Hip *z*-score (mean)		Total Spine (L1–L4) *z*-score (mean)	
		Pre	Post	Pre	Post
0–3 ^c^	1	2.4	2.2	1.4	1.1
>3–6	4	1.2	1.0	−0.1	−0.1
>6–12	1	0.8	0.7	0.0	0.2
>12	2	−0.2	0.1	−1.6	−1.4
All	8	0.5	0.5	−0.2	−0.1
Range		−0.6 to 3.2	−0.4 to 3.0	−2.9 to 1.4	−2.3 to 1.1

Normal Values: Normal BMD > −1.0; ^a^ The women participated in a 6-month carbohydrate-protein (CHO-PRO) Supplement intervention (360 kcal/day); ^b^ At pre-intervention, the women completed a self-reported menstrual history. Based on this questionnaire, this is the number of months between starting the intervention and their last menstrual cycle; ^c^ All women in the intervention were amenorrheic except for this individual, who was classified as oligomenorrheic.

### 3.5. Muscle Power and Torque and Markers of Protein Synthesis and Degradation

**Figure 2:**
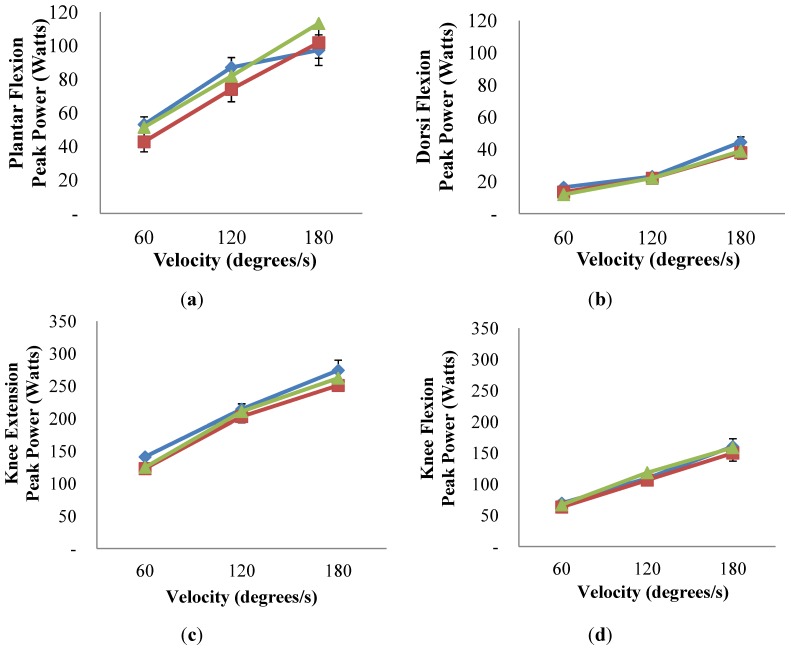
Peak Power for plantar flexion (*i.e.*, ankle extension) (**a**); dorsi flexion (*i.e.*, ankle flexion) (**b**); knee flexion (**c**); and knee extension (**d**) during isokinentic contractions in Eumenorrheic and ExMD women at pre/post-intervention ^a^.

^a^ Eumenorrheic (Eumen) were measured at baseline (0-month; ) only and compared to ExMD at 0-month () and at 6-months (). No differences were detected (*p*-value > 0.05 for all comparisons). Multiple linear regression was used to compare Eumen and ExMD groups and 0-month *vs.* 6-months in ExMD group, blocking on participant and controlling for baseline age and weight and false discovery rate (FDR) of 5%.

Peak power (Figure 2) and average power (data not shown) for ankle extension and flexion and knee flexion and extension during isokinetic contractions did not change with the intervention in ExMD group (*p* > 0.05). Explosive power (data not shown) also did not improve with the intervention, and both groups had similar peak, average, and explosive power (*p* > 0.05).

Peak power (Figure 2) and average power (data not shown) for ankle extension and flexion and knee flexion and extension during isokinetic contractions did not change with the intervention in ExMD group (*p* > 0.05). Explosive power (data not shown) also did not improve with the intervention, and both groups had similar peak, average, and explosive power (*p* > 0.05). Phosphorylated states of AMPK (energy status indicator), p70S6K (protein synthesis marker) and FOXO1 (protein degradation marker were not altered by the intervention or different between group (data not shown; *p* > 0.05).

### 3.6. Profile of Mood States

**Figure 3:**
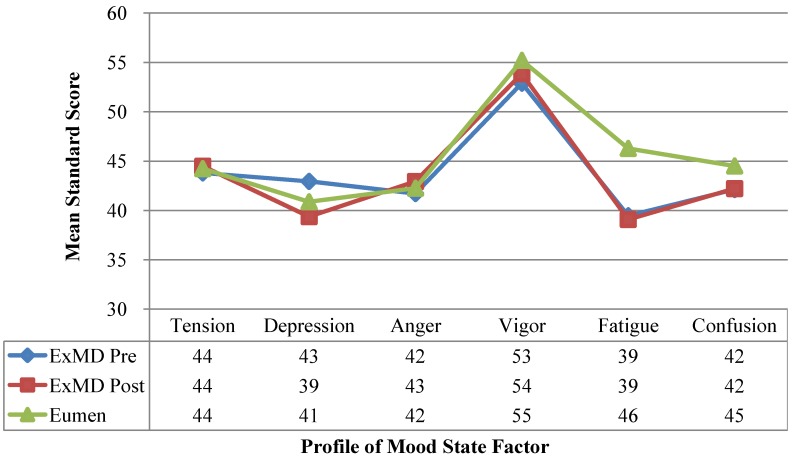
Profile of Mood State Profile in women with Eumenorrheic and ExMD at pre/post-intervention. Please provide the original figure format, like Figure 2.

^a^ Eumenorrheic (Eumen) were measured at baseline (0-month) only and compared to ExMD at 0-month and at 6-months. No differences were detected (*p*-value > 0.05 for all comparisons). Multiple linear regression was used to compare Eumen and ExMD groups and 0-month *vs.* 6-months in ExMD group, blocking on participant and controlling for baseline age and weight and false discovery rate (FDR) of 5%.

Total mean POMS or domain scores were not different between groups at baseline or 6-months (Figure 3). Mean fatigue scores were 14% lower in the ExMD *vs.* Eumen, but differences were not significant (*p* = 0.17). With the intervention, overall mood state did not change.

## 4. Discussion

This is the first study to demonstrate that when ExMD athletes maintain exercise training and consistently consume an extra 360 kcal/day for 6-months, menses and ovulation (all but one) are restored. These findings are consistent with results reported by Mallinson *et al.* [32] where menstrual status was restored in two amenorrheic athletes with increased EI (mean increase in EI was 276 kcal/day for one athlete and 1881 kcal/day for the other) and Kopp-Woodroffe *et al.* [18] where menses and ovulation was restored in three amenorrheic athletes during a 20–24 week diet (360 kcal/day) and exercise intervention [18]. To improve overall compliance, we only altered EI and did not include 1 day/week of rest to reduced EEE as used by Kopp-Woodroffe *et al.* [18]. Some women and/or their coaches are reluctant to add rest days to their training programs. With the intervention, we observed improvements in EA and EB, and EB neared balance. However, these increases were not statistically significant, which was most likely due to the large fluctuations in EI observed in two participants during the intervention. EI will fluctuate as athletes move in and out of their competitive season, which occurred during the 6-month intervention. For the ExMD group, mean baseline EA was higher than those reported by Loucks *et al.* [33] in sedentary women (30 kcal/kg FFM) experiencing disruption of LH pulsatility. Our ExMD participants did, however, resume menses at EA values similar to those reported in sedentary women (45 kcal/kg FFM) with normal LH pulsatility [33]. The observed discrepancy in EA threshold between the present study and Loucks *et al.* [33] is likely due primarily to the subject differences. Specifically, we examined free-living physically active women with ExMD, while Loucks *et al.* [33] used an acute (4 day) energy restriction protocol to disrupt LH pulsatility in sedentary women in a clinical research setting. Thus, there may not be a set EA threshold that induces chronic ExMD in female athletes, with some more sensitive to changes in EA and develop ExMD at a higher level of EI.

### 4.1. Energy and Nutrient Intakes

Energy and macronutrients intakes did not different between groups at baseline. These data are similar to others who examined physically active women without eating disorders [15]. In amenorrheic athletes, fat intake (g/day) has been reported to be lower compared to Eumen [15]. We also observed a lower fat intake (17 g/day lower) in ExMD *vs.* Eumen, but these differences were not statistically significant. Finally, consistent with previous research [34,35], we found no differences in bone-related micronutrients between our groups (ExMD/Eumen).

### 4.2. Menstrual Status and Blood Hormone

Reproductive hormones were similar between groups and did not change with the intervention. We did not expect to observe a difference in these hormones since they were measured in the early follicular phase (1–5 day after the onset of menses). Except for estradiol, reproductive hormones have been reported to be similar between ExMD and Eumen athletes [18,36,37], while three studies report higher estradiol levels [38,39,40]. The timing of hormonal assessment can impact results with differences more likely to be detected when measured later in the menstrual cycle (2–9 days after the onset of menses) [38]. Similar to others [2,41], we also found no differences in thyroid levels between groups, while Zanker and Swaine reported higher levels in Eumen *vs.* ExMD [39,40].

### 4.3. Bone Health

The ExMD had similar BMD for whole body, total hip, and spine at pre/post-intervention *vs.* the Eumen group. Our findings are similar to others who have measured whole body [34] and hip BMD [34,42,43,44] in athletes using blood hormones to confirm ExMD status. However, many studies reported lower spinal BMD in ExMD runners *vs.* Eumen controls [34,35,42,43,44]. These differences could be due to duration of ExMD and whether individuals with disordered eating were eliminated. Overall, duration of ExMD varied from >1.7 to 6.5 year [34,35,44], while others do not report duration [42,43]. Conversely, only two of our athletes had ExMD >1 year; Nattiv *et al.* [2] reported a decline in BMD with increased number of missed menstrual cycles. We also carefully screened for and eliminated athletes with disordered eating, which few studies do [42,43]. Disordered eating has been associated with low BMD in women with normal menstrual status [2,3]. Taken together, the discrepancies in spinal BMD findings are likely due to these experimental design choices.

Spinal BMD and BMC were improved in two ExMD participants post-intervention, but no change in mean total BMD was observed. Case [45] and observational [46] studies have reported improvements in spinal BMD that corresponded to weight gain, while others [47] report spinal BMD improvements independent of weight gain. More time may be required after resumption of menses (*i.e.*, ≥1 year) to detect significant improvements in BMD and BMC. Another confounding factor is age. Berger *et al*. [48] reported that peak BMD occurred between 33 and 40 year in the lumbar spine and between 16 and 19 year in the total hip. Consistent with this, two women in our study had low hip BMD and realized no improvements with the intervention. Improvements in spinal BMD, however, were realized in one ExMD women with low BMD and one with osteoporosis. Thus, improvements observed in BMC could be due to change in menstrual status and age.

### 4.4. Blood Bone Markers

We found no differences in serum bone formation/resorption markers between groups, and no change with the intervention. Most studies reported no difference in serum osteocalcin levels between ExMD and Eumen physically active women [44,49,50,51]. Only, Zanker and Swaine found lower osteocalcin levels in amenorrheic *vs.* Eumen runners [40]. To our knowledge, only De Souza *et al.* [49] has reported lower P1NP in women with hypothalamic amenorrhea (HPA)/low energy status compared to both women with HPA/normal energy status or Eumen women. In their study, low energy status was defined as a RMR ratio of actual *vs.* predicted that is <0.09 using Harris-Benedict equation [49]. Based on this definition, all the women our study would have been classified with normal energy status. We found similar P1NP levels in our ExMD to those reported by De Souza *et al.* [49] for women with HPA/normal energy status; there were no differences between ExMD and Eumen groups. For urinary CTX, De Souza *et al.* [49] reported similar levels in physically active women with/without menses, which is consistent with our findings. Urinary and serum CTX measures are reported to correspond [52].

### 4.5. Muscle Torque and Power and Markers of Protein Synthesis and Degradation

To our knowledge, this is the first study to examine the impact of ExMD on muscle strength and power. Contrary to our hypothesis, we found no difference in muscle strength and power with resumption of menses. Our findings are consistent with reports that knee flexion and extension torque was similar during high and low estrogen phases of menstrual cycle in eumenorrheic trained females [53,54,55,56,57]. Only Phillips *et al.* [58] reported a relationship between torque and menstrual cycle phase. The applicability of these finding to athletic performance, however, is questionable since measurements were only made in the adductor pollicis (*i.e.*, thumb) [58]. Of the studies that reported no differences in torque, knee flexion and extension was measured at 60 degree/s in three studies [53,56,57], at 120 degree/s in one study [55], and at 180 degree/s in two studies [56,57]. Both the ExMD and Eumen women in the present study had lower mean knee flexion and extension torque at slower speeds (60 and 120 degree/s) but greater values at faster speeds (180 degree/s) than previously reported in eumenorrheic women [53,54,55,56,57]. A plausible explanation for these differences could be the athletic background of the participants, but only Hertel *et al.* [55] specified the types of athletes included. In addition, muscle assessments in the present study were made upon completion of a 6-month intervention. At this time the women in ExMD group had only experienced on average ~3 months of normal menstrual cycles. It is plausible that a longer time period after resumption of menses is required before improvements in muscle strength and health are realized.

We also expected that low EA and estradiol levels would negatively alter protein synthesis and degradation markers (*i.e.*, AMPK for energy status, p70S6K for protein synthesis, and FOXO1 for protein degradation). Contrary to our hypothesis, we found no differences in phosphorylated AMPK, p70S6K, and FOXO1 between the Eumen and ExMD groups and due to the intervention. Our findings are consistent with results reported in eumenorrheic women by Miller *et al.* [14] where post-exercise protein synthesis was similar in luteal and follicular phase of menstrual cycle after consuming a CHO-PRO drink (64% CHO, 15% PRO, 21% fat) every 30-min for 4-h post-exercise (cycling for 60 min at 67% maximal watts). The present study, however, examined the impact of chronically low estrogen levels (*i.e.*, ExMD) and low EA on markers of protein synthesis utilizing a different protocol (*i.e.*, the women ingested only a single CHO-PRO drink immediately after running for 45-min at 75% VO_2_max with markers assessed at 1-h post-exercise). Further research examining the impact of ExMD on muscle strength and power and post-exercise muscle protein metabolism is warranted.

### 4.6. Profile of Mood State

Overall, the POMS profile of both groups was similar to the “iceberg profile” reported by Morgan [59,60] and the POMS profile of athletes (*n* = 2086) reported by Terry and Lane [61]. Compared to Terry and Lane, our ExMD participants had lower fatigue scores (pre/post), similar depression scores (pre), and lower depression scores (post). All scores, except vigor, were below norm scores for college-aged population. Based on the observed trends, ExMD athletes may have depressed sensitivity to fatigue, thus, reducing their drive to eat and producing a lower EI.

### 4.7. Strengths and Limitations

Intervention compliance was monitored carefully with weekly meeting. We also carefully screened for reproductive disorders besides ExMD, but could only evaluate reproductive hormones with the single blood draw at pre/post. Repeated pulsatile hormonal assessments would have strengthened the study, but were not available. We used EB as our key variable for the calculation of power, which indicated that a sample size of 8 participants was needed. A larger sample size would have increased statistical power. However, it is difficult to get female athletes to commit to such a long intervention. The study required participants’ involvement over approximately a 10-month period, including pre/post-assessments and the 6-month intervention. Self-reported EI estimates should be interpreted with caution; however, we did carefully trained participants to weigh/record food over a 7 day training period, screened for underreporting, and eliminated those with eating disorders. The larger than predicted variability in EI was due to two participants and contributed to our inability to detect statistically significant changes in energy-related variables. Our estimates of TEE were improved by measured RMR and RunEE and the use of accelerometers to monitor training volume. Also, the daily supplement provided energy and macro/micronutrients. Finally, multiple comparisons were made in the same group of individuals; the probability of a false positive increases with multiple comparisons. We did, however, control for this statistically using FDR adjustments.

## 5. Conclusions

We demonstrated that a 6-month intervention that provided an extra energy (+360 kcal/day) and resulting in moderate weight gain (1.6 kg) could successfully reverse ExMD. We also observed important physiological increases in EI (+382 kcal/day) and EA (+417 kcal/day) and nearly normalizing EB, which coincided with reversal of ExMD; however, these changes were not statistically significant. We found no difference in EA and EB between groups at baseline, suggesting that some female athletes may be more sensitive to fluctuations in these variables. Women with longer duration of ExMD took more time to resume menses and had lower BMD. Based on this study, a dietary invention that improves EA and EB, while minimizing fat gain, could be an appropriate non-pharmacological approach to treat ExMD.

## References

[1] Rodriguez N.R., di Marco N.M., Langley S. (2009). American college of sports medicine position stand. Nutrition and athletic performance. Med. Sci. Sports Exerc..

[2] Nattiv A., Loucks A.B., Manore M.M., Sanborn C.F., Sundgot-Borgen J., Warren M.P., American College of Sports Medicine (2007). American college of sports medicine position stand. The female athlete triad. Med. Sci. Sports Exerc..

[3] Mountjoy M., Sundgot-Borgen J., Burke L., Carter S., Constantini N., Lebrun C., Meyer N., Sherman R., Steffen K., Budgett R. (2014). The ioc consensus statement: Beyond the female athlete triad--relative energy deficiency in sport (red-s). Br. J. Sports Med..

[4] Gibbs J.C., Williams N.I., de Souza M.J. (2013). Prevalence of individual and combined components of the female athlete triad. Med. Sci. Sports Exerc..

[5] Warren M.P., Perlroth N.E. (2001). The effects of intense exercise on the female reproductive system. J. Endocrinol..

[6] Nattiv A., Kennedy G., Barrack M.T., Abdelkerim A., Goolsby M.A., Arends J.C., Seeger L.L. (2013). Correlation of mri grading of bone stress injuries with clinical risk factors and return to play: A 5-year prospective study in collegiate track and field athletes. Am. J. Sports Med..

[7] Hoch A.Z., Lal S., Jurva J.W., Gutterman D.D. (2007). The female athlete triad and cardiovascular dysfunction. Phys. Med. Rehabil. Clin. N. Am..

[8] Enns D.L., Tiidus P.M. (2010). The influence of estrogen on skeletal muscle: Sex matters. Sports Med..

[9] Enns D.L., Iqbal S., Tiidus P.M. (2008). Oestrogen receptors mediate oestrogen-induced increases in post-exercise rat skeletal muscle satellite cells. Acta Physiol. (Oxf.).

[10] Carter A., Dobridge J., Hackney A.C. (2001). Influence of estrogen on markers of muscle tissue damage following eccentric exercise. Fiziol. Cheloveka.

[11] Kanaley J.A., Ji L.L. (1991). Antioxidant enzyme activity during prolonged exercie in amenorrheic and eumenorrheic athletes. Metab. Clin. Exp..

[12] Sipaviciene S., Daniuseviciute L., Kliziene I., Kamandulis S., Skurvydas A. (2013). Effects of estrogen fluctuation during the menstrual cycle on the response to stretch-shortening exercise in females. BioMed. Res. Int..

[13] Markofski M.M., Braun W.A. (2014). Influence of menstrual cycle on indices of contraction-induced muscle damage. J. Strength Cond. Res..

[14] Miller B.F., Hansen M., Olesen J.L., Flyvbjerg A., Schwarz P., Babraj J.A., Smith K., Rennie M.J., Kjaer M. (2006). No effect of menstrual cycle on myofibrillar and connective tissue protein synthesis in contracting skeletal muscle. Am. J. Physiol. Endocrinol. Metab..

[15] Manore M.M., Kam L.C., Loucks A.B., International Association of Athletics Federations (2007). The female athlete triad: Components, nutrition issues, and health consequences. J. Sports Sci..

[16] American Society of Health-System Pharmacists, Inc. Estrogen and Progestin (Oral Contraceptives). http://www.nlm.nih.gov/medlineplus/druginfo/meds/a601050.html.

[17] Arends J.C., Cheung M.Y., Barrack M.T., Nattiv A. (2012). Restoration of menses with nonpharmacologic therapy in college athletes with menstrual disturbances: A 5-year retrospective study. Int. J. Sport Nutr. Exerc. Metab..

[18] Kopp-Woodroffe S.A., Manore M.M., Dueck C.A., Skinner J.S., Matt K.S. (1999). Energy and nutrient status of amenorrheic athletes participating in a diet and exercise training intervention program. Int. J. Sport Nutr..

[19] Garner D.M., Olmstead M.P., Polivy J. (1983). Development and validation of a multidimensional eating disorder inventory for anorexia nervosa and bulimia. Int. J. Eat. Disord..

[20] Sundgot-Borgen J. (1993). Nutrient intake of female elite athletes suffering from eating disorders. Int. J. Sport Nutr..

[21] McNair D.M., Lorr M., Droppleman L.F. (1992). Revised Manual for the Profile of Mood States.

[22] Practice Committee of the American Society for Reproductive Medicine (2008). Current evaluation of amenorrhea. Fertil. Steril..

[23] Guebels C.P., Kam L.C., Maddalozzo G.F., Manore M.M. (2014). Active women before/after an intervention designed to restore menstrual function: Resting metabolic rate and comparison of four methods to quantify energy expenditure and energy availability. Int. J. Sport Nutr. Exerc. Metab..

[24] Woolf K., LoBuono D.L., Manore M.M., Beals K.A. (2013). B-vitamins and physical activity: Is need increased?. Nutrition and the Female Athlete.

[25] Willis K.S., Smith D.T., Broughton K.S., Larson-Meyer D.E. (2012). Vitamin d status and biomarkers of inflammation in runners. Open Access J. Sports Med..

[26] Seibel M.J. (2005). Biochemical markers of bone turnover: Part I: Biochemistry and variability. Clin. Biochem. Rev..

[27] Tomten S.E., Hostmark A.T. (2006). Energy balance in weight stable athletes with and without menstrual disorders. Scand. J. Med. Sci. Sports.

[28] Goldberg G.R., Black A.E., Jebb S.A., Cole T.J., Murgatroyd P.R., Coward W.A., Prentice A.M. (1991). Critical evaluation of energy intake data using fundamental principles of energy physiology: 1. Derivation of cut-off limits to identify under-recording. Eur. J. Clin. Nutr..

[29] Writing Group for the ISCD Position Development Conference (2004). Diagnosis of osteoporosis in men, premenopausal women, and children. J. Clin. Densitom..

[30] Drouin J.M., Valovich-mcLeod T.C., Shultz S.J., Gansneder B.M., Perrin D.H. (2004). Reliability and validity of the biodex system 3 pro isokinetic dynamometer velocity, torque and position measurements. Eur. J. Appl. Physiol..

[31] Bassey E.J., Short A.H. (1990). A new method for measuring power output in a single leg extension: Feasibility, reliability and validity. Eur. J. Appl. Physiol. Occup. Physiol..

[32] Mallinson R.J., Williams N.I., Olmsted M.P., Scheid J.L., Riddle E.S., de Souza M.J. (2013). A case report of recovery of menstrual function following a nutritional intervention in two exercising women with amenorrhea of varying duration. J. Int. Soc. Sports Nutr..

[33] Loucks A.B., Verdun M., Heath E.M. (1998). Low energy availability, not stress of exercise, alters l h pulsatility in exercising women. J. Appl. Physiol..

[34] Gremion G., Rizzoli R., Slosman D., Theintz G., Bonjour J.P. (2001). Oligo-amenorrheic long-distance runners may lose more bone in spine than in femur. Med. Sci. Sports Exerc..

[35] Rencken M.L., Chesnut C.H., Drinkwater B.L. (1996). Bone density at multiple skeletal sites in amenorrheic athletes. JAMA.

[36] De Souza M.J., Maguire M.S., Maresh C.M., Kraemer W.J., Rubin K.R., Loucks A.B. (1991). Adrenal activation and the prolactin response to exercise in eumenorrheic and amenorrheic runners. J. Appl. Physiol..

[37] Wilmore J.H., Wambsgans K.C., Brenner M., Broeder C.E., Paijmans I., Volpe J.A., Wilmore K.M. (1992). Is there energy conservation in amenorrheic compared with eumenorrheic distance runners?. J. Appl. Physiol..

[38] Glass A.R., Deuster P.A., Kyle S.B., Yahiro J.A., Vigersky R.A., Schoomaker E.B. (1987). Amenorrhea in olympic marathon runners. Fertil. Steril..

[39] Zanker C.L., Swaine I.L. (1998). Relation between bone turnover, oestradiol, and energy balance in women distance runners. Br. J. Sports Med..

[40] Zanker C.L., Swaine I.L. (1998). Bone turnover in amenorrhoeic and eumenorrhoeic women distance runners. Scand. J. Med. Sci. Sports.

[41] Loucks A.B. (2004). Energy balance and body composition in sports and exercise. J. Sports Sci..

[42] Snead D.B., Stubbs C.C., Weltman J.Y., Evans W.S., Veldhuis J.D., Rogol A.D., Teates C.D., Weltman A. (1992). Dietary patterns, eating behaviors, and bone mineral density in women runners. Am. J. Clin. Nutr..

[43] Snead D.B., Weltman A., Weltman J.Y., Evans W.S., Veldhuis J.D., Varma M.M., Teates C.D., Dowling E.A., Rogol A.D. (1992). Reproductive hormones and bone mineral density in women runners. J. Appl. Physiol..

[44] Stacey E., Korkia P., Hukkanen M.V., Polak J.M., Rutherford O.M. (1998). Decreased nitric oxide levels and bone turnover in amenorrheic athletes with spinal osteopenia. J. Clin. Endocrinol. Metab..

[45] Fredericson M., Kent K. (2005). Normalization of bone density in a previously amenorrheic runner with osteoporosis. Med. Sci. Sports Exerc..

[46] Drinkwater B.L., Nilson K., Ott S., Chesnut C.H. (1986). Bone mineral density after resumption of menses in amenorrheic athletes. JAMA.

[47] Zanker C.L., Cooke C.B., Truscott J.G., Oldroyd B., Jacobs H.S. (2004). Annual changes of bone density over 12 years in an amenorrheic athlete. Med. Sci. Sports Exerc..

[48] Berger C., Goltzman D., Langsetmo L., Joseph L., Jackson S., Kreiger N., Tenenhouse A., Davison K.S., Josse R.G., Prior J.C. (2010). Peak bone mass from longitudinal data: Implications for the prevalence, pathophysiology, and diagnosis of osteoporosis. J. Bone Miner. Res..

[49] De Souza M.J., West S.L., Jamal S.A., Hawker G.A., Gundberg C.M., Williams N.I. (2008). The presence of both an energy deficiency and estrogen deficiency exacerbate alterations of bone metabolism in exercising women. Bone.

[50] Gibson J.H., Mitchell A., Harries M.G., Reeve J. (2004). Nutritional and exercise-related determinants of bone density in elite female runners. Osteoporos. Int..

[51] Pettersson U., Stalnacke B., Ahlenius G., Henriksson-Larsen K., Lorentzon R. (1999). Low bone mass density at multiple skeletal sites, including the appendicular skeleton in amenorrheic runners. Calcif. Tissue Int..

[52] Seibel M.J., Robins S.P., Bilezikian J.P. (2006). Dynamics of bone and cartilage metabolism.

[53] Janse de Jonge X.A., Boot C.R., Thom J.M., Ruell P.A., Thompson M.W. (2001). The influence of menstrual cycle phase on skeletal muscle contractile characteristics in humans. J. Physiol..

[54] Lebrun C.M., McKenzie D.C., Prior J.C., Taunton J.E. (1995). Effects of menstrual cycle phase on athletic performance. Med. Sci. Sports Exerc..

[55] Hertel J., Williams N.I., Olmsted-Kramer L.C., Leidy H.J., Putukian M. (2006). Neuromuscular performance and knee laxity do not change across the menstrual cycle in female athletes. Knee Surg. Sports Traumatol. Arthrosc..

[56] DiBrezzo R., Fort I.L., Brown B. (1991). Relationships among strength, endurance, weight and body fat during three phases of the menstrual cycle. J. Sports Med. Phy. Fit..

[57] Abt J.P., Sell T.C., Laudner K.G., McCrory J.L., Loucks T.L., Berga S.L., Lephart S.M. (2007). Neuromuscular and biomechanical characteristics do not vary across the menstrual cycle. Knee Surg. Sports Traumatol. Arthrosc..

[58] Phillips S.K., Sanderson A.G., Birch K., Bruce S.A., Woledge R.C. (1996). Changes in maximal voluntary force of human adductor pollicis muscle during the menstrual cycle. J. Physiol..

[59] Hallas J., Bjerrum L., Stovring H., Andersen M. (2008). Use of a prescribed ephedrine/caffeine combination and the risk of serious cardiovascular events: A registry-based case-crossover study. Am. J. Epidemiol..

[60] Smith D., Bar-Eli M. (2007). Essential Readings in Sport and Exercise Psychology.

[61] Terry P.C., Lane A.M. (2000). Normative values for the profile of mood states for use with athletic samples. J. Appl. Sport Psychol..

